# Brain Network Changes in Lumbar Disc Herniation Induced Chronic Nerve Roots Compression Syndromes

**DOI:** 10.1155/2022/7912410

**Published:** 2022-05-14

**Authors:** Yan-Peng Zhang, Guang-Hui Hong, Chuan-Yin Zhang

**Affiliations:** ^1^Department of Orthopedics, Renhe Hospital, Baoshan District, Shanghai 200431, China; ^2^Department of Orthopedics, Affiliated Renhe Hospital of Shanghai University, Shanghai 200431, China; ^3^Department of Orthopedics, The First Affiliated Hospital of Fujian Medical University, Fujian 350005, China

## Abstract

Lumbar disc herniation (LDH) induced nerve compression syndromes have been a prevalent problem with complex neural mechanisms. Changes in distributed brain areas are involved in the occurrence and persistence of syndromes. The present study aimed to investigate the changes of brain functional network in LDH patients with chronic sciatica using graph theory analysis. A total of thirty LDH adults presenting L4 and/or L5 root (s) compression syndromes (LDH group) and thirty age-, sex-, BMI- and education-matched healthy control (HC group) were recruited for functional MRI scan. Whole-brain functional network was constructed for each participant using Pearson's correlation. Global and nodal properties were calculated and compared between two groups, including small-worldness index, clustering coefficient, characteristic path length, degree centrality (*DC*), betweenness centrality (*BC*) and nodal efficiency. Both LDH and HC groups showed small-world architecture in the functional network of brain. However, LDH group showed that nodal centralities (*DC*, *BC* and nodal efficiency) increased in opercular part of inferior frontal gyrus; and decreased in orbital part of inferior frontal gyrus, lingual cortex and inferior occipital gyrus. The DC and efficiency in the right inferior occipital gyrus were negatively related with the Oswestry Disability Index in LDH group. In conclusion, the LDH-related chronic sciatica syndromes may induce regional brain alterations involving self-referential, emotional responses and pain regulation functions. But the whole-brain small-world architecture was not significantly disturbed. It may provide new insights into LDH patients with radicular symptoms from new perspectives.

## 1. Introduction

Lumbar disc herniation (LDH) is a prevalent disease caused by degenerative pathologies of lumbar intervertebral disc. Low back pain is the most common symptom with a high occurrence rate and great burden of cost [1–3]. Researches have revealed corresponding changes in the brain associated with chronic low back pain using functional magnetic resonance imaging (fMRI). Changes involved specific brain regions, functional connectivity, and properties of whole-brain network [4–9]. These studies have greatly broadened our insight into low back pain related brain alterations. However, low back pain is a highly prevalent symptom with obscure causes in most cases. Only in a minority of cases does it directly links to some defined organic disease exist. It may not be necessarily causally related with LDH neither [10–12]. Even in LDH patients who complain of low back pain, the causes for low back pain may still be unclear. In clinical practice, it is not uncommon that LDH patients merely report nerve compression symptoms without obvious existence of low back pain.

In clinical practice, many LDH patients would develop persistent radicular symptoms, while some even need to receive surgical treatment of decompression and lumbar fusion. However, not all patients reported satisfactory relief of neuropathic pain even following appropriate treatments [13, 14]. It is acknowledged that pathological changes in the peripheral nerve would lead to complex disorders involving both peripheral and central nervous systems [15–17]. Except for the factors of peripheral nerve, maladaptive changes in the brain may also contribute to the failure of symptom relief. However, brain changes related with sciatica due to herniated nucleus pulposus have not been revealed yet. The present study aimed to explore the changes of brain at whole-brain network level specifically associated with chronic unilateral nerve root (s) compression in LDH patients by functional magnetic resonance image (fMRI). We restricted LDH patients to those who chiefly complained of radicular symptoms that affecting only one leg at the time of recruitment. Graph theoretical analysis was applied to characterize the functional connectivity between each pair of brain regions [18]. Global and nodal properties of the functional brain network were calculated to quantitively describe the network and differences of the properties were compared between LDH-induced sciatica patients and healthy controls.

## 2. Materials and Methods

### 2.1. Participants

LDH induced nerve root(s) compression patients (LDH group) were recruited according to the following inclusion criteria: (1) lumbar disc herniation diagnosed by MRI assessment; (2) chiefly complained of unilateral sciatica symptoms for at least 3 months; (3) unilateral L4 and/or L5 nerve root(s) compression confirmed by clinical symptoms [positive in straight leg raising test (SLRT)], physical examination and electrophysiological tests; (4) aged 18 or more, no gender limitation; (5) unilateral nerve compression symptoms persisted for at least 3 months; (6) no other pain except for LDH related pain; (7) no neurological deficiencies, such as visual or hearing loss; (8) no abnormal findings, such as infarction or focal lesion in brain MRI presentation, confirmed by two blinded independent radiologists. Exclusion criteria: (1) with contraindications or inability to tolerate MR scan; (2) with neurological disease or brain lesions, such as traumatic brain injury, stroke, neurodegenerative disease, brain tumor, epilepsy; (2) with psychiatric diseases, such as schizophrenia, depression, anxiety disorder before the onset of current LDH-induced radiculopathy; (3) reported a history of drug abuse or alcohol addiction; (4) for other reasons they were unsuitable to undergo MR scan.

Inclusion criteria for healthy controls (HC group): (1) aged 18 or more, no gender limitation; (2) generally healthy without a record of chronic systemic disease (e.g. diabetes mellitus, hypertension); (3) no record of neurological or psychiatric disorders, such as stroke, depression, or epilepsy; (4) no neurological deficiencies, such as visual or hearing loss; (5) no abnormal findings, such as infarction or focal lesion in brain MRI presentation, confirmed by two blinded independent radiologists. Exclusion criteria: (1) with contraindications or inability to tolerate MR scan; (2) with a history of alcohol/drug addiction; (3) unwilling to participate in the present study or already enrolled in another trial.

This study was approved by the Institutional Review Board of Renhe Hospital (No. KJ2019-06). All the participants provided written consents.

### 2.2. Clinical Assessments

Oswestry Disability Index (ODI) and Visual Analogue Scale (VAS) were measured in LDH patients. ODI was the standard for evaluating the severity of LDH. The score of ODI ranges from 0 to 100, with higher scores indicating more impaired function [19]. VAS measures the amount of pain that a patient feels, ranges from 0 to 10, indicating from none to extreme amount of pain [19]. It was evaluated both at rest and during SLRT (recorded at 60° of hip flexion on the symptomatic side; recorded as 10 points if the patient was unable to achieve 60°).

### 2.3. MR Image Acquisition

MR images were acquired with a 3.0 T MRI scanner (Siemens Verio, Erlangen, Germany). During the scan, the participants were instructed to lie still and relax with their eyes open. Foam pad and earplugs were applied to limit head motion and reduce the impact of machine noise. They were asked to keep awake and not to think about anything in particular. Resting-state fMRI were acquired axially with an echo-planar imaging (EPI) sequence according to the following protocol: repetition time (TR) =3000 ms, echo time (TE) = 30 ms, flip angle = 90°, field of view (FOV) = 240 × 240 mm^2^, resolution = 64 × 64 matrix, slice number = 43, slice thickness = 3 mm, voxel size = 3.75 × 3.75 × 3 mm^3^, gap = 0, number of volume = 240. Three-dimensional T1-weighted images were acquired with magnetization-prepared rapid gradient echo (MPRAGE) sequence with following parameters: repetition time (TR) = 1900 ms, echo time (TE) = 2.93 ms, inversion time = 900 ms, flip angle = 9°, resolution = 256 × 256 matrix, slice number = 160, slice thickness = 1.0 mm, voxel size = 1 × 1 × 1 mm^3^.

### 2.4. Data Preprocessing

The brain images were preprocessed with the Data Processing Assistant for Resting-State fMRI (DPARSF) toolbox, which was based on Matlab, SPM12 (https://www.fil.ion.ucl.ac.uk/spm), and DPABI [20]. In patients with right leg affected, brains were left-to-right flipped so that the affected side of brain were localized to one side of the hemisphere [21, 22]. The first 10 volumes of functional images were discarded to allow for adaptation to the signal stabilization. The remaining 190 volumes were corrected for different slice acquisition times with the middle image of each repetition time (TR) as reference. Head motion with the Friston 24-parameter method and nuisance signals of white matter, cerebrospinal fluid and head-motion parameters were regressed out [23]. Structural T1 images were segmented for coregisteration of functional images, which were then normalized to the Montreal Neurological Institute (MNI) space using Diffeomorphic Anatomical Registration Through Exponentiated Lie Algebra (DARTEL). The functional images were resampled to 3 mm isotropic voxels and spatially smoothed with 6 mm full width at half maximum (FWHM) Gaussian kernel. The normalized function images were temporally filtered with 0.01-0.1 Hz bandpass to reduce physiological noises and low frequency drifting. Images with head motion of >3 mm translation or> 3°rotation were excluded from the study.

### 2.5. Network Construction and Graph Theoretical Analysis

The cortical and subcortical areas of the whole brain were parcellated into 90 regions of interest (ROIs) based on the prior atlas of Anatomical Automatic Labeling (AAL) [24]. These 90 ROIs were defined as nodes of the network. The mean time series of each ROI was extracted and the correlation between each pair of nodes represented edges. The correlations coefficient (r-value) between each pair of ROIs was calculated with Pearson's correlation measure. The r-values were transformed to z-values with Fisher's z transformation to obtain near-normally-distributed data. An adjacency matrix of z-values was constructed for each subject and binary undirected connectivity network was then obtained with selected thresholds (sparsity). The sparsity was set from 10% to 46% with an interval step of 0.01 [25]. Topological properties of the network were calculated based on the network.

### 2.6. Network Properties

Global properties included: clustering coefficient (*C_p_*), characteristic path length (*L_p_*), normalized clustering coefficient (*γ*), normalized characteristic path length (*λ*) and small-worldness (*σ*). Nodal properties included: degree centrality (*DC*), betweenness centrality (*BC*), and efficiency (*E*) of a given node (Detailed definition, equations and clinical implications of these properties have been included in the Supplemental Materials (Supplementary Table S1) [26, 27]. The functional networks of whole brain were constructed with GRETNA (v2.0.0) toolbox (https://www.nitrc.org/projects/gretna) [28].

### 2.7. Statistical Analysis

Two-sample t-test and Chi-squared test were applied in the comparison of demographic characteristics between two groups. The area under the curve (AUC) of properties and nodal properties between two groups were compared with nonparametric permutation tests [29]. The significance level was set at *p* < 0.05 in the analysis of global properties, while in nodal properties set at *p* < 0.05 after false discovery rate (FDR) correction for multiple comparison.

### 2.8. Correlation Analysis

For properties showing significant between-group differences, partial correlation analysis was performed between these properties and clinical variable (ODI and VAS) in the LDH group. The effects of age and sex were controlled. Software SPSS (V21, SPSS Inc., Chicago, IL, USA) was used.

## 3. Results

### 3.1. Characteristics of Participants

Thirty LDH induced nerve compression patients (LDH group) (56.3 ± 9.7 yrs) and thirty HC subjects (HC group) (55.0 ± 12.3 yrs) were enrolled in the analysis (Table 1). Age, sex, body mass index (BMI), and education level (low education: junior middle school or below; high education: senior high school or higher) were comparable between two groups (all *p* > 0.05). In the LDH group, L4 nerve root was involved in six patients, L5 in twenty patients, while both L4 and L5 were involved in the rest four patients. LDH patients received conservative treatments, including oral medicine (neurotrophic drug on a regular basis; NSAIDS or opiates as needed), lying flat, physical therapy, lumbar traction or traditional Chinese treatments (e.g. acupuncture, herbal medicine). But these patients still complained of radicular symptoms at enrollment.

### 3.2. Clinical Assessments

All patients reported moderate or severe symptoms with unilateral side involved. Left side was affected in seventeen patients, while right side in the rest thirteen patients. The average ODI score was 65.93 ± 11.46. All our patients were able to achieve 60 degree of hip flexion during SLRT. The VAS score was 3.67 ± 0.99 at rest and 6.67 ± 0.96 during SLRT, respectively.

### 3.3. Global Properties

**Figure 1:**
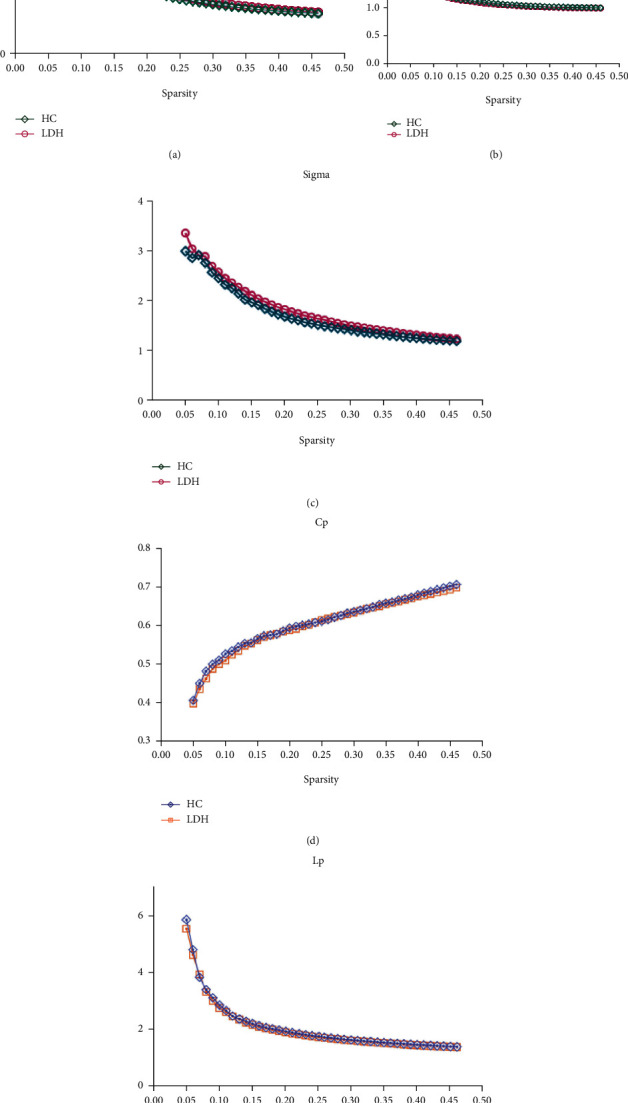
Changes of small-world parameters, clustering coefficient (C_p_) and characteristic path length (L_p_) in the lumbar disc herniation induce nerve root compression patients and healthy controls as sparsity ranged from 0.1 to 0.46. No significant difference was found between two groups in the normalized clustering coefficients (*γ*) (a), normalized characteristic path length (*λ*) (b). small-worldness (*σ*) (C), clustering coefficient (D) and characteristic path length (E) over a sparsity range of 0.1-0.46 (all p > 0.05).

Over the sparsity range of 0.05-0.46, both LDH and HC groups showed small-world topology of functional network, which was characterized by normalized clustering coefficient (*γ*)> > 1, normalized characteristic path length (*λ*) ≈ 1, and small-worldness (*σ*) = *γ*/*λ* > 1. No significant difference was found in *γ*, *λ*, *σ*, C_p_, and L_p_ by comparing area under curve (AUC) between groups (all *p* > 0.05) (Figure 1). Results without flipping procedure see Supplemental Materials (Supplementary Figure S1).

### 3.4. Nodal Properties

**Figure 2:**
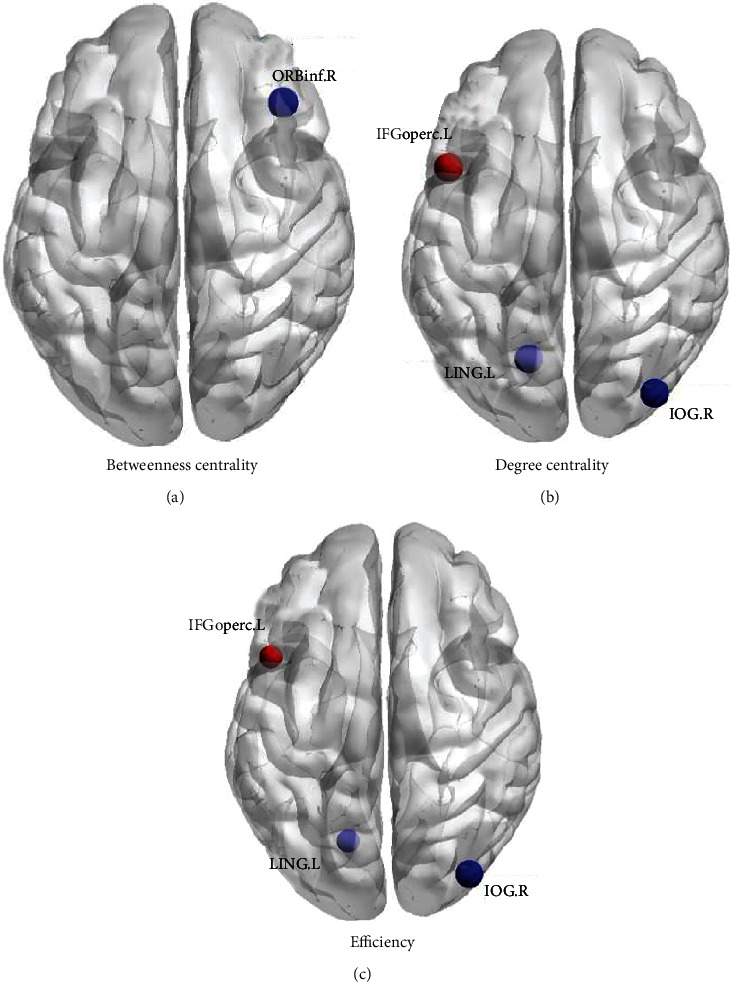
Differences of betweenness centrality (*BC*) (A), degree centrality (*DC*) (B) and efficiency (*E*) (C) of a node between lumbar disc herniation (LDH) induced nerve root(s) compression patients and healthy control subjects. The red balls represent increased values of nodal properties in the LDH group while the blue balls represent decreased, compared with the healthy control (HC) group. The size of ball represents significance, with bigger balls indicating smaller *p*-values. R: right; L: left; ORBinf: inferior frontal gyrus, orbital part; IFGoperc: inferior frontal gyrus, opercular part; LING: lingual gyrus; IOG: inferior occipital gyrus.

Compared with HCs, the LDH group showed increased betweenness centrality (*BC*) in right inferior frontal gyrus (orbital part) (ORBinf.R); and no decreased *BC* (Figure 2(a), Table 2). LDH group showed increased degree centrality (*DC*) in left inferior frontal gyrus, opercular part (IFGoperc.L); and decreased *DC* in left lingual gyrus (LING.L) and right inferior occipital gyrus (IOG.R) (Figure 2(b), Table 3). Nodal efficiency increased in left inferior frontal gyrus, opercular part (IFGoperc.L); and decreased in left lingual gyrs (LING.L) and right inferior occipital gyrus (IOG.R) (Figure 2(c), Table 4). Results without flipping procedure see Supplemental Materials (Supplementary Figure S2, Supplementary Tables S2–4).

### 3.5. Correlation Analysis

**Figure 3:**
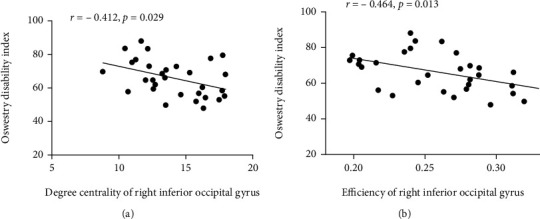
Correlation between Owestry Disability Index and degree centrality and efficiency of right inferior occipital gyrus. Partial correlation analysis indicated positive correlation between Owestry Disability Index and degree centrality of right inferior occipital gyrus (*r* = -0.412, *p* = 0.029), as well as between Owestry Disability Index and efficiency of right inferior occipital gyrus (*r* = -0.464, *p* = 0.013) while controlling the effects of age and sex.

Significant partial correlation was found between DC in IOG. R and ODI (*r* = -0.412, *p* = 0.029), E in IOG.R and ODI (*r* = -0.464, *p* = 0.013) (Figure 3). No significant correlation was found in the rest network properties showing significant between-group differences and clinical variables.

## 4. Discussion

LDH is a common pathology that causes a series of symptoms, including chronic low back pain, radicular leg pain, weakness, and paresthesia in affected area [30, 31]. As the brain is capable of adapting to abnormal physical status, complicated changes would occur in the brain associated with LDH induced symptoms. Accumulating researches have also provided evidence that pathology-specific brain alterations play a crucial role in the occurrence and maintenance of pain, especially chronic pain [32–36]. Previous studies have confirmed the involvement of brain plasticity in LDH patients. Shishi et al. demonstrated whole-brain network disruption in degree, clustering coefficient, and efficiency in patients with LDH-related chronic pain (including chronic low back pain and/or leg pain), indicating decreased hubness, segregation and integration [5]. According to Jing et al., individuals with low back pain due to LDH showed significantly longer characteristic path length and lower clustering coefficient, global efficiency and local efficiency. They also showed decreased functional connectivity in several brain regions including anterior cingulate cortex, middle cingulate cortex, et al [6]. However, they did not restrict investigations to LDH patients with unilateral radicular symptoms related with nerve root(s) compression.

The present study focused on patients with chronic radicular symptoms induced by LDH, which has not been investigated yet. Specifically, graph theory was applied in the analysis of resting-state fMRI data. Both LDH-induced sciatica patients and HC showed small-world architecture in the functional brain network at the global level. No significant change of global properties was found in LDH group under a wide range of sparsity thresholds. A small-world network is supposed to be more efficient in information transfer either than the regular or the random network. It was used to characterize a balance between global and local efficiency of information transfer [18, 37]. Therefore, the properties of small-world were not significantly interrupted in these LDH-induced sciatica patients under selected range of sparsities. The results implied that these patients still showed efficient small-world architecture, which was optimal balance of segregation and integration. Human brain is a complex interconnected network. Although 1-2 lumbar nerve roots were in pathological status, the whole brain still organized well in a large scale. It may be due to the capability of the whole brain network to compensate the disturbance of local peripheral nerve roots.

However, abnormal nodal properties were still found in several brain regions in LDH group compared with healthy controls. The nodal properties BC, DC and nodal efficiency represents the importance of a given node in the regional or global network. Specifically, BC characterizes the effect of a given node on information flow between other nodes; DC reflects the information communication ability of a node in the functional network; the nodal efficiency characterizes the efficiency of parallel information transfer of that node in the network.

In the present study, nodal centralities increased in a node located in the frontoparietal network (IFGoperc.L); while decreased in the default mode network (DMN) (ORBinf.R) and visual network (lingual gyrus and inferior occipital gyrus) [38]. The brain DMN composes of a wide range of brain regions, primarily including medial prefrontal cortex (mPFC), posterior cingulate cortex (PCC), and inferior parietal lobule (IPL) [39, 40]. It describes the baseline state of neural activities in the brain which is related with self-related mental activities [41]. Increase in regional neural activity and functional connectivity in DMN were found related with depression [42, 43]. Occipital cortex regions, including calcarine fissure and lingual gyrus were noted showing LDH-related decrease in nodal centralities. According to literature reviewer, lingual gyrus was also involved in the process of pain [44, 45]. Therefore, the present study indicated strengthened coordinating role of several brain regions in these patients, involving functions of self-referential activities and pain modulation. Nodal centralities increase was found in the LDH group in regions within the frontoparietal network (IFGoperc.L) [38]. Frontoparietal network and its functional connectivity with insula were related with emotion regulation [46]. Increased nodal centralities in this region suggested potential compensation in LDH-induced sciatica patients on pain and emotional regulation functions. It may also be a compensation to the abnormal signal afferent of peripheral nerve in pathological status such as nerve compression syndromes. However, the exact relationship between abnormal nodal properties and psychomental status need further explorations. Future studies involving specific scales that representing different aspects of the mind status would be worthwhile.

In the correlation analysis, after controlling the effects of age and sex, we only noted significant negative correlation between ODI and DC in IOG.R, E in IOG.R. No significant correlation was found between clinical variables and other abnormal network properties in the LDH group. It implied correlation of altered nodal properties in IOG.R with clinical performance.

There are still several issues need to be further addressed in future studies. A larger sample size of participants need to be included in future researches to enhance the power of statistics. Altered properties related with intervention and its predictive effects in prognoses also need exploration. The structure network and its relation with functional network would be an important supplement to this field. However, the present study still brought us deeper insights into brain functioning in LDH-induced radiculopathy form the aspect of network. Brain regions with significantly changed local properties can be potential targets for future intervention, such as neuromodulation.

## 5. Limitations

As it was an observational study comparing LDH patients with healthy controls, randomization was inapplicable in the assignment of participants. While the confounding factors were controlled, there might be potential bias due to subject selection. As the effects of oral medicine were inevitable in the present clinical study, further researches (eg, experimental studies) with confounding factors controlled are needed to provide supplement evidence.

## 6. Conclusions

The present study constructed functional network of brain by resting-state fMRI for lumbar disc herniation (LDH) induced chronic nerve root compression patients and healthy controls. Graph theoretical analysis was used to investigate global and local properties of the brain. Both groups showed small-world architecture but no between-group difference was found in small-world measures. The LDH groups exhibited decreased nodal centralities in nodes of limbic, ventral attentional and frontoparietal networks; while increased nodal centralities mainly in nodes of default mode and visual networks. The study provided greater insights into brain network alterations related with LDH-induced chronic sciatica syndromes. A better understanding of brain plasticity may help us in decision making regarding the treatment strategies such as oral medication or surgery. Finally, the sample size was relatively small. In our future work, investigations with a larger sample size are still needed to draw a more convincing conclusion.

## Figures and Tables

**Table 1:** Demographic characteristic of the lumbar disc hernia induced unilateral lumbar nerve root compression and healthy control groups.

Characteristics	LDH (n = 30)	HC (n = 30)	*p*-value
Male sex - no. (%)	19 (63.3%)	17 (56.7%)	0.598^a^
Age -yr	56.3 ± 9.7	55.0 ± 12.3	0.635^b^
BMI – Kg/m^2^	23.46 ± 3.07	22.27 ± 2.39	0.099^b^
Education			
Low education	10	14	0.292^a^
High education	20	16	
Affected root(s)			
L4	6	—	—
L4 and L5	4	—	—
L5	20	—	—
Affected side			
Left	17	—	—
Right	13	—	—
Duration (month)	5.4 ± 2.4	—	
ODI	65.93 ± 11.46	—	
VAS at rest	3.67 ± 0.99	—	
VAS during SLRT	6.67 ± 0.96	—	

LDH: lumbar disc herniation; HC: healthy control; BMI: body mass index; ODI: Oswestry Disability Index; VAS: Visual Analogous Scale; SLRT: Straight Leg Raising Test. Low education: junior middle school or below; high education: senior high school or higher. ^a^Chi-square test; ^b^two-sample t-test.

**Table 2:** Brain regions with significant different betweenness centrality (BC) between LDH induced nerve root(s) compression and healthy control groups.

Brain region	*p-*value (uncorrected)
HC > LDH	
ORBinf.R	0.001

LDH: lumbar disc herniation; HC: healthy control; R: right; ORBinf: inferior frontal gyrus, orbital part.

**Table 3:** Brain regions with significant different degree centrality (DC) between LDH induced nerve root(s) compression and healthy control groups.

Brain region	*p*-value (uncorrected)
HC > LDH	
LING.L	0.001
IOG.R	0.001
LDH > HC	
IFGoperc.L	0.001

LDH: lumbar disc herniation; HC: healthy control; R: right; L: left; LING: lingual gyrus; IOG: inferior occipital gyrus; IFGoperc: inferior frontal gyrus, opercular part.

**Table 4:** Brain regions with significant different efficiency of a given node between LDH induced nerve root(s) compression and healthy control groups.

Brain region	*p*-value (uncorrected)
HC > LDH	
LING.L	0.003
IOG.R	0.001
LDH > HC	
IFGoperc.L	0.003

LDH: lumbar disc herniation; HC: healthy control; R: right; L: left; LING: lingual gyrus; IOG: inferior occipital gyrus; IFGoperc: inferior frontal gyrus, opercular part.

## Data Availability

The data of the present study would be available from the corresponding author upon request.

## Supplementary Materials

Supplementary Materials Figure S1: Changes of small-world parameters, clustering coefficient (Cp) and characteristic path length (Lp) in the lumbar disc herniation induce nerve root compression patients and healthy controls as sparsity ranged from 0.1 to 0.46. Figure S2: Differences of betweenness centrality (BC), degree centrality (DC) and efficiency between lumbar disc herniation (LDH) induced nerve root(s) compression patients and healthy control subjects. Table S1: The definition, equation and clinical implication of network properties. Table S2: Brain regions with significant different betweenness centrality (BC) between LDH induced nerve root(s) compression and healthy control groups. Table S3: Brain regions with significant different degree centrality (DC) between LDH induced nerve root(s) compression and healthy control groups. Table S4: Brain regions with significant different efficiency (E) of a given node between LDH induced nerve root(s) compression and healthy control groups.
