# Supplementary material for: Brain Network Changes in Lumbar Disc Herniation Induced Chronic Nerve Roots Compression Syndromes
Source: Neural Plast. 2022 May 14;2022:7912410. doi: 10.1155/2022/7912410 (PMC9124092; doi:10.1155/2022/7912410)
Supplement: Supplementary Materials — Figure S1: Changes of small-world parameters, clustering coefficient (Cp) and characteristic path length (Lp) in the lumbar disc herniation induce nerve root compression patients and healthy controls as sparsity ranged from 0.1 to 0.46. Figure S2: Differences of betweenness centrality (BC), degree centrality (DC) and efficiency between lumbar disc herniation (LDH) induced nerve root(s) compression patients and healthy control subjects. Table S1: The definition, equation and clinical implication of network properties. Table S2: Brain regions with significant different betweenness centrality (BC) between LDH induced nerve root(s) compression and healthy control groups. Table S3: Brain regions with significant different degree centrality (DC) between LDH induced nerve root(s) compression and healthy control groups. Table S4: Brain regions with significant different efficiency (E) of a given node between LDH induced nerve root(s) compression and healthy control groups. [file 7912410.f1.docx]

**Supplementary Materials**

Content

[Figure S1 2](#_Toc102205557)

[Figure S2 3](#_Toc102205558)

[Table S1 4](#_Toc102205559)

[Table S2 5](#_Toc102205560)

[Table S3 6](#_Toc102205561)

[Table S4 7](#_Toc102205562)

# Figure S1


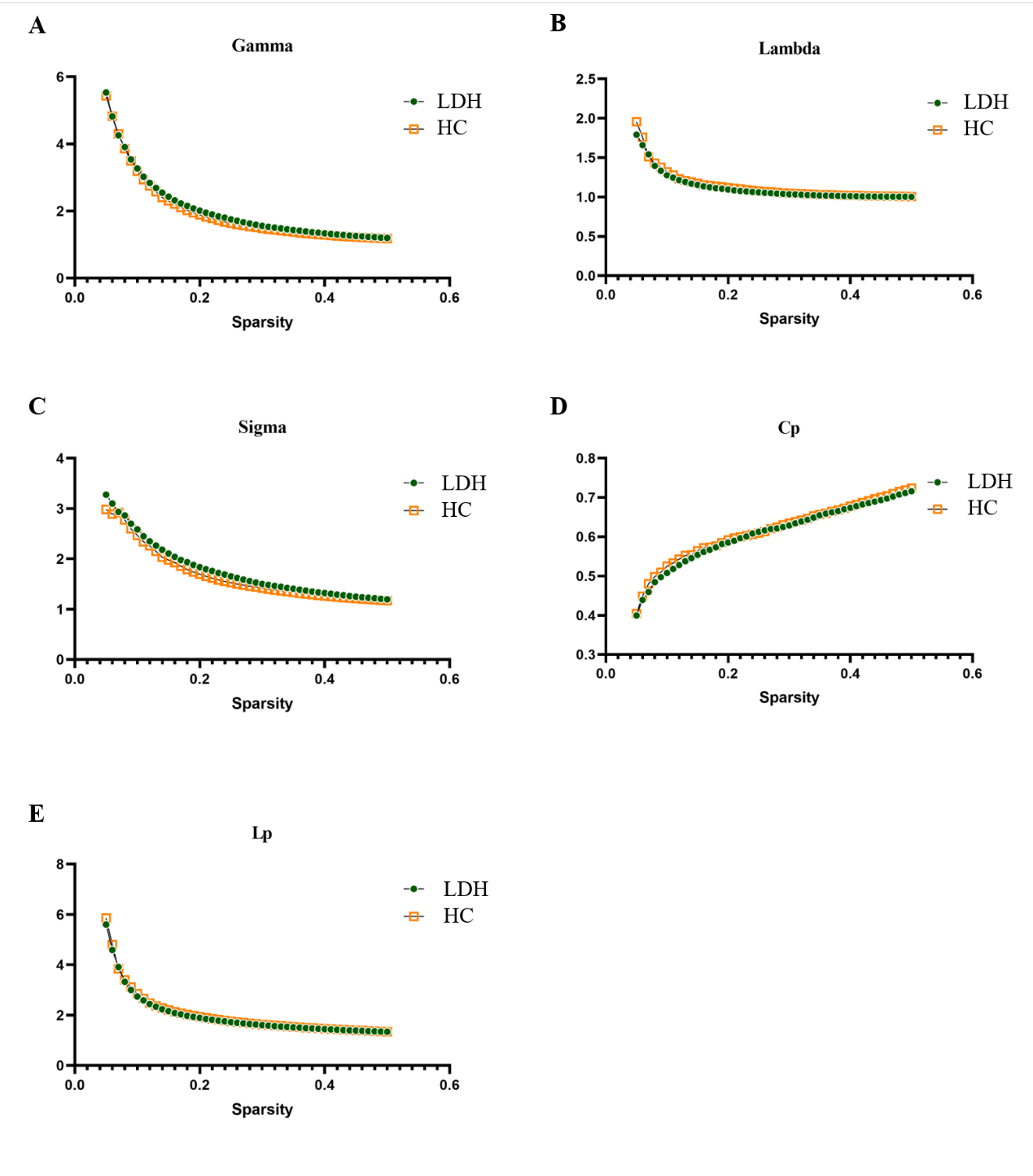


Figure S1. Changes of small-world parameters, clustering coefficient (*C_p_*) and characteristic path length (*L_p_*) in the lumbar disc herniation induce nerve root compression patients and healthy controls as sparsity ranged from 0.1 to 0.46. No significant difference was found between two groups in the normalized clustering coefficients (*γ*) (A), normalized characteristic path length (*λ*) (B). small-worldness (*σ*) (C), clustering coefficient (D) and characteristic path length (E) over a sparsity range of 0.1-0.46 (all p>0.05). *LDH: lumbar disc herniation; HC: healthy control.*

# Figure S2


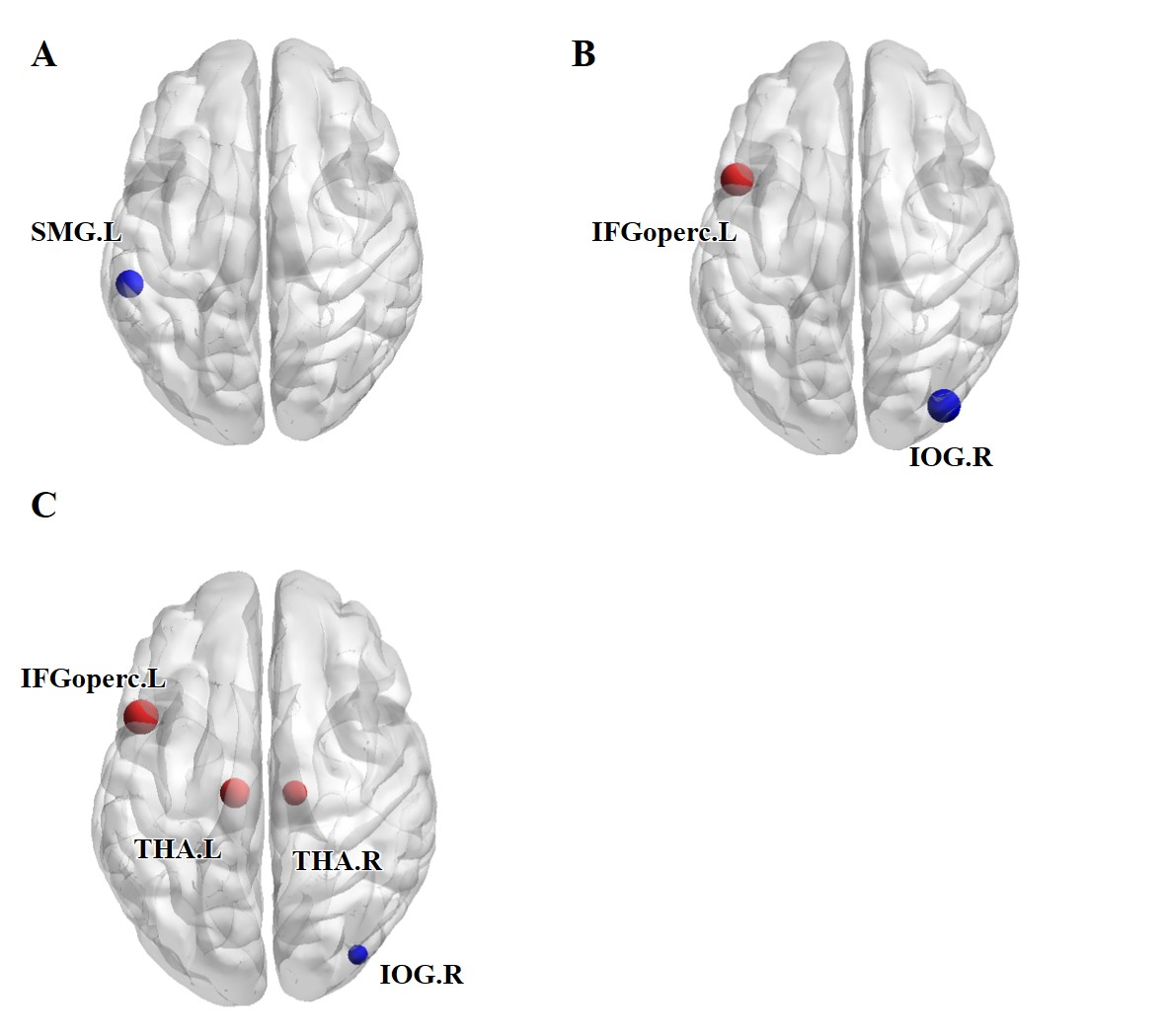


Figure S2. Differences of betweenness centrality (*BC*) (A), degree centrality (*DC*) (B) and efficiency (*E*) (C) between lumbar disc herniation (LDH) induced nerve root(s) compression patients and healthy control subjects. The red balls represent increased values of nodal properties in the LDH group while the blue balls represent decreased, compared with the healthy control (HC) group. The size of ball represents significance, with bigger balls indicating smaller p-values.

*R: right; L: left; SMG: supramarginal gyrus; IFGoperc: inferior frontal gyrus, opercular part; THA: thalamus; IOG: inferior occipital gyrus.*

# Table S1

Table S1. The definition, equation and clinical implication of network properties.

| Network properties | Definition | Equation | Clinical implication |
| --- | --- | --- | --- |
| Clustering coefficient  (*Cp*) of a network | *Cp* of a node is defined as the ratio of number of existing edges to the number of all possible edges in the node’s direct neighbors. *Cp* of a network is the mean of *Cp* over all nodes in the network. | $C_{p}\left( G \right)=\frac{1}{N}\sum_{i=1}^{N} \frac{{2a}_{i}}{k_{i}\left( k_{i}-1 \right)}$, *k_i_* is the degree of node i, and *a_i_* is the number of edges between the ki neighbors of node *i*, N represents the number of nodes in graph G. | *Cp* quantifies the local interconnectivity of graph. |
| Characteristic path length (*Lp*) of a network | *Lp* of a network is the mean shortest path length over all pair of nodes in the network. | $L_{p}\left( G \right)=\frac{1}{N(N-1)}\sum_{i\neq j\in N} L_{ij}$, where *L_ij_* is the shortest path length between node *i* and node *j* in graph *G*, N represents the number of nodes in a network. | *Lp* is an indicator of overall routing efficiency of graph. |
| Small-worldness index (*σ*) | *σ* is the ratio of normalized *Cp* (*γ*) and normalized *Lp* (*λ*). | $\sigma=\frac{\gamma}{\lambda}= \frac{\frac{Cp}{{Cp}_{rand}}}{\frac{Lp}{{Lp}_{rand}}}$, where *Cp* and *Cp_rand_* are clustering coefficient, and *Lp* and *Lp_rand_* are characteristic path length of real-world network and random networks, respectively. | If *σ* is greater than 1, the network can be considered as a small-world network which is characterized by minimum path length between any pair of nodes and greater local interconnectivity. |
| Betweenness centrality (*BC*)  of a given node | The fraction of all shortest paths in the network that passed through a given node. | $BC\left( i \right)=\sum_{i\neq j\neq k\in G} \frac{L_{jk}(i)}{L_{jk}}$, where *L_jk_* is the number of the shortest paths between node *j* and node *k*, and *L_jk_* (*i*) is the number of shortest paths that transverse node *i*. | *BC* identifies nodes which play a “bridge spanning” role in a network, and detect important anatomical or functional connections. |
| Degree centrality (*DC*) of a given node | The ratio of the number direct connections between a given node and other nodes to the number of all possible connections. | $DC\left( i \right)=\frac{1}{N_{i}}\sum_{i\neq j\in N} a_{ij}$, where *a_ij_* indicates direct connections between node *i* and node *j*, N represents the number of nodes in a network, N_i_ represents all possible connections between node i and other nodes. | It is most commonly used as a measure of density, or the total “wiring cost” of the network. Nodes with a high DC are interacting, structurally or functionally, with many other nodes in a network. |
| Efficiency (*E*) of a given node | Efficiency of a given node is defined as the mean of the inverse of the shortest path length between the node and all of the other nodes. | $E\left( i \right)=\frac{1}{N-1}\sum_{i\neq j\in G} \frac{1}{L_{ij}}$, where *L_ij_* is the shortest path length between node *i* and node *j* in graph *G*, N represents the number of nodes in a network. | Efficiency of a given node measures the global efficiency of the parallel information exchange in graph *G.* |

# Table S2

Table S2. Brain regions with significant different betweenness centrality (*BC*) between LDH induced nerve root(s) compression and healthy control groups

| Brain region | *p-*value (uncorrected) |
| --- | --- |
| HC>LDH |  |
| SMG.L | 0.003 |

*LDH: lumbar disc herniation; HC: healthy control; R: right; SMG: supramarginal gyrus.*

# Table S3

Table S3. Brain regions with significant different degree centrality (*DC*) between LDH induced nerve root(s) compression and healthy control groups

| Brain region | *p*-value (uncorrected) |
| --- | --- |
| HC>LDH |  |
| IOG.R | 0.002 |
| LDH>HC |  |
| IFGoperc.L | 0.002 |

*LDH: lumbar disc herniation; HC: healthy control; R: right; L: left;* *IOG: inferior occipital gyrus;* *IFGoperc: inferior frontal gyrus, opercular part.*

# Table S4

Table S4. Brain regions with significant different efficiency (*E*) of a given node between LDH induced nerve root(s) compression and healthy control groups

| Brain region | *p-*value (uncorrected) |
| --- | --- |
| HC>LDH |  |
| IOG.R | 0.004 |
| LDH>HC |  |
| IFGoperc.L | 0.001 |
| THA.L | 0.002 |
| THA.R | 0.003 |

*LDH: lumbar disc herniation; HC: healthy control; R: right; L: left; IOG: inferior occipital gyrus;* *IFGoperc: inferior frontal gyrus, opercular part; THA: thalamus.*
